# Supplementary material for: Identification of TLR2 Signalling Mechanisms Which Contribute to Barrett’s and Oesophageal Adenocarcinoma Disease Progression
Source: Cancers (Basel). 2021 Apr 25;13(9):2065. doi: 10.3390/cancers13092065 (PMC8123271; doi:10.3390/cancers13092065)
Supplement: Supplementary file 1 [file cancers-13-02065-s001.zip › cancers-1187975-supplementary.pdf]

cancers

MDPI

Article

Identification of TLR2 Signalling Mechanisms Which Contribute to Barrett's and Oesophageal Adenocarcinoma Disease Progression

Ewelina Flis [1], Gillian Barber [1], Ciara Nulty [1], Brian Keogh [1], Peter McGuirk [1], Akanksha Anand [2], Jacintha O'Sullivan [3], Michael Quante [2] and Emma M. Creagh [1,*]

[1] School of Biochemistry and Immunology, Trinity Biomedical Science Institute (TBSI), Trinity College Dublin, D02 R590 Dublin, Ireland; flise@tcd.ie (E.F.); barberg@tcd.ie (G.B.); nultyci@tcd.ie (C.N.); keogh.brian@nuritas.com (B.K.); mcguirk.peter@gmail.com (P.M.)
[2] Department of Internal Medicine, Technical University of Munich, D-80333 Munich, Germany; akanksha.anand@tum.de (A.A.); michael.quante@tum.de (M.Q.)
[3] Department of Surgery, Trinity Translational Medicine Institute, Trinity Centre for Health Sciences, St. James's Hospital, D08 W9RT Dublin, Ireland; osullij4@tcd.ie
* Correspondence: ECREAGH@tcd.ie; Tel.: +353-1-8962539

Citation: Flis, E.; Barber, G.; Nulty, C.; Keogh, B.; McGuirk, P.; Anand, A.; O'Sullivan, J.; Quante, M.; Creagh, E.M. Identification of TLR2 Signalling Mechanisms Which Contribute to Barrett's and Oesophageal Adenocarcinoma Disease Progression. *Cancers* **2021**, *13*, 2065. https://doi.org/10.3390/cancers13092065

Academic Editor: Axel Hillmer

Received: 1 April 2021
Accepted: 22 April 2021
Published: 25 April 2021

Publisher's Note: MDPI stays neutral with regard to jurisdictional claims in published maps and institutional affiliations.

Copyright: © 2021 by the authors. Licensee MDPI, Basel, Switzerland. This article is an open access article distributed under the terms and conditions of the Creative Commons Attribution (CC BY) license (http://creativecommons.org/licenses/by/4.0/).

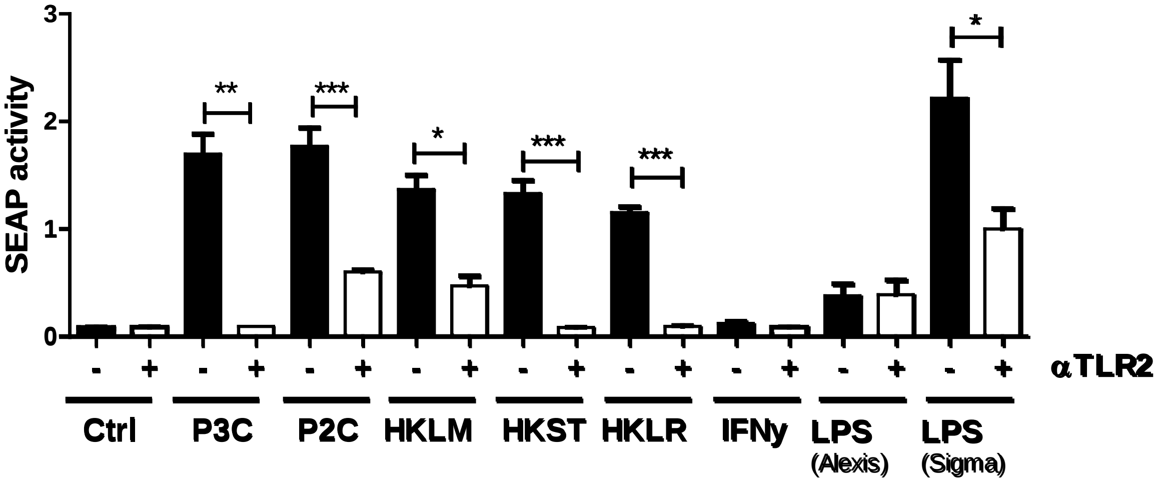

Figure S1. Direct TLR2 stimulation of the TLR reporter cell line. THP1-XBlue-CD14, is inhibited by the neutralising antibody, αTLR2. THP1-XBlue-CD14 were left untreated or pre-treated for 1 h with αTLR2 (10 µg/ml) prior to stimulation with P3C (0.05 µg/ml, Invivogen, tlrl-pms); P2C (0.05 µg/ml, Invivogen, tlrl-pm2s-1); heat-killed bacteria: *Listeria monocytogenes* (HKLM, 10^7 cells/ml, Invivogen, tlrl-hklm); *Salmonella typhimurium* (HKST, 10^6 cells/ml, Invivogen, tlrl-hkst2); *Lactobacillus rhamnosus* (HKLR, 10^7 cells/ml, Invivogen, tlrl-hklr); IFNγ (40 ng/ml, PeproTech, 300-02); LPS from *Escherichia coli*, Serotype R515 (Alexis, TLRGRADE, 1 µg/ml, ALX-581-007-L001) or LPS from *Escherichia coli* O111:B4 (Sigma, 1 µg/ml, L4391) for 24 h. TLR stimulation was assessed by measuring supernatant levels of SEAP using QUANTI-Blue reagent. Values represent the mean ± SEM of three independent experiments. Statistical analysis was performed using unpaired 2-tailed Student t-test to compare mean values between αTLR2 treated and untreated cells, *$p < 0.05$; ** $p < 0.01$; ***$p < 0.001$.

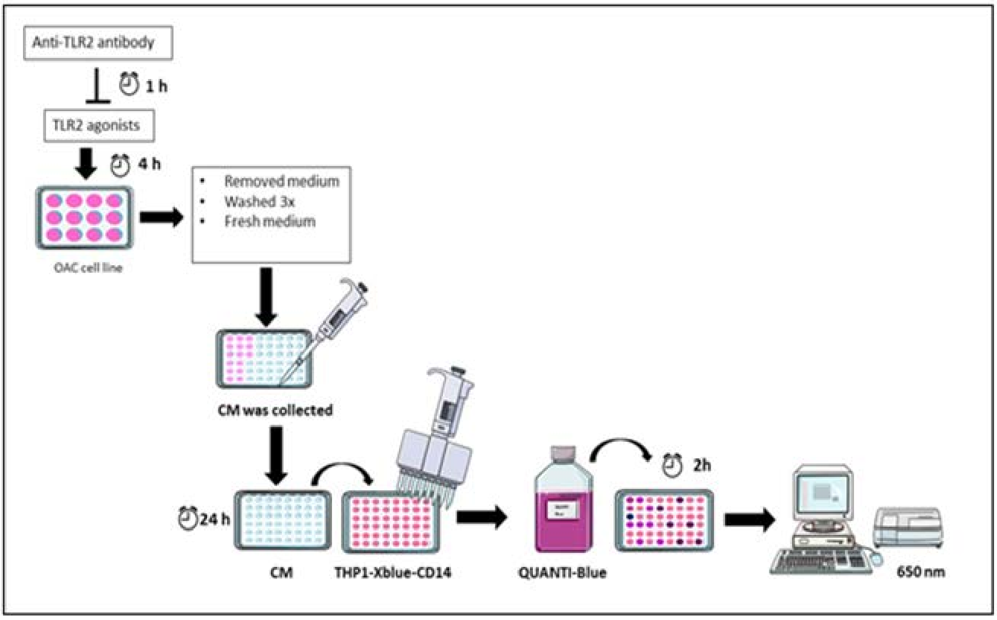

Figure S2. Experimental scheme**.** Oesophageal cell lines (GO, SK-GT 4 and OE33) (seeded at 2×10^5, 1.6×10^5, 2×10^5 cell/mL, respectively) were left unstimulated or stimulated with αTLR2 (10 µg/mL) for 1 h prior to stimulation with P3C (0.05 µg) / P2C (0.05 µg)/ or LPS (1 µg/mL) for 4 h Media were removed, cells were washed ×3 with PBS and fresh media was added/well. After 24 h, 50 µL CM was collected and added to 50 µL THP1-XBlue-CD14 (seeded at 4×10^6 cells/mL) and incubated for 24 h at 37°C. 40 µL resulting supernatant was added to 160 µL QUANTI Blue™ and incubated for 2 h at RT in a 96 well plate prior to absorbance measurement at 650 nm.

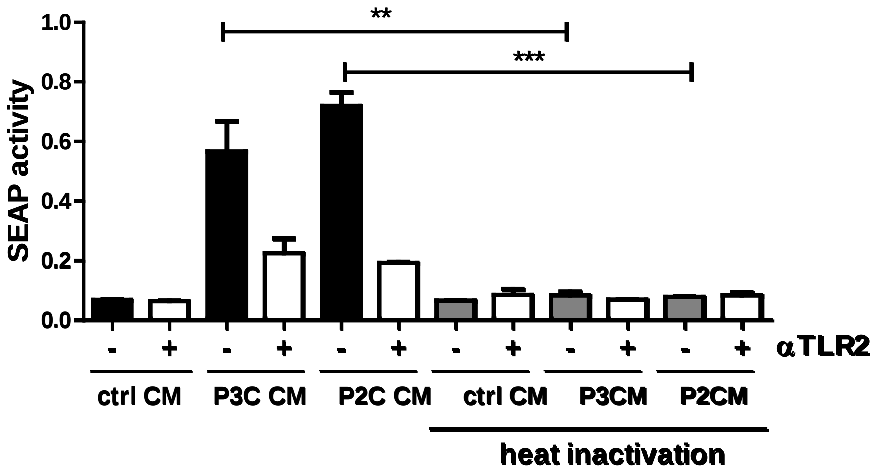

Figure S3. Heat-inactivated CM is unable to activate TLRs**.** CM was collected from unstimulated or αTLR2 stimulated SK-GT4 (ctrl CM), P2C-pretreated SK-GT 4 (P2C CM) and P3C-pretreated SK-GT4 (P3C CM). Half of the collected CM were heat-inactivated by incubating for 1 h at 75° C. 50 µL ctrl CM, P3C CM, P2C CM and heat-inactivated ctrl CM, P3C CM and P2C CM were added to THP1-Xblue-CD14 and incubated for 24 h at 37°C. 40 µL supernatant was added to 160 µL QUANTI-Blue™ and incubated for 2 h at RT in a 96 well plate prior to absorbance measurement at 650nm. Values represent the mean ± SEM of two independent experiments (run in. duplicate). Statistical analysis was performed using 2-way *ANOVA* test to compare mean values between CM and heat-inactivated CM, ** $p < 0.01$;*** $p < 0.001$.
